# Robot-Assisted Gait Training for Achieving Gait Symmetry in a Subacute Stroke Patient: A Case Report

**DOI:** 10.7759/cureus.75361

**Published:** 2024-12-09

**Authors:** Ren Fujii, Makoto Tamari, Naomichi Mizuta, Naruhito Hasui, Yuki Nonaka

**Affiliations:** 1 Department of Rehabilitation, Musashigaoka Hospital, Kumamoto, JPN; 2 Department of Physical Therapy, Reiwa Health Science University, Fukuoka, JPN; 3 Department of Rehabilitation, Nihon Fukushi University, Handa, JPN; 4 Depertment of Therapy, Takarazuka Rehabilitation Hospital, Takarazuka, JPN

**Keywords:** gait asymmetry, gait pattern, gait training, robot, stroke

## Abstract

Gait asymmetry in post-stroke patients is an important gait characteristic that is associated with their balance control, inefficiency, and risks of musculoskeletal injury to the non-paretic lower limb and falling. Unfortunately, most stroke patients retain an asymmetrical gait pattern, even though their gait independence and gait speed improve. We describe the clinical course of a subacute stroke patient who achieved a symmetrical gait at discharge after undergoing both gait training with orthoses and robot-assisted gait training from the early intervention phase. A Korean woman in her 50s developed a right frontal subcortical hemorrhage. She had severe left upper- and lower-extremity motor paralysis and was unable to walk independently. Her gait pattern was also observed to have a knee extension thrust pattern and a resulting asymmetric gait pattern. The gait interventions consisted of gait training with a knee-ankle-foot orthosis (KAFO) and an ankle-foot orthosis (AFO), in addition to robot-assisted gait training from the early onset. The control of the knee joint's movement was obtained by the attachment of the knee-ankle-foot robot to the paretic lower limb. Following these interventions, the patient was able to walk independently and had a symmetrical gait pattern at the time of discharge. The combination of robot-assisted gait training and gait training with orthoses for subacute stroke patients, as is widely used in general populations, may prevent the patients' mislearning of gait movements and contribute to the acquisition of a symmetrical gait pattern.

## Introduction

The recovery of gait performance is a major goal of stroke rehabilitation [1]. In earlier studies, approximately 50% of stroke patients were able to walk independently after rehabilitation, but the remaining ~50% retained gait asymmetry [2]. Gait asymmetry is defined by spatial asymmetry (i.e., the step length on the paretic/non-paretic side) and temporal asymmetry (i.e., the stance or swing phase time on the paretic/non-paretic side) [3], and gait asymmetry affects an individual's balance control, inefficiency, and the risks of musculoskeletal injury to the non-paretic lower limb and falling [2,3]. Gait performance is an important variable in stroke rehabilitation, but the optimal type of rehabilitation to improve stroke patients' gait performance is not well established.

Various gait-training devices have been developed and were shown to improve the abnormal gait patterns of stroke patients [4]. Gait rehabilitation robots are representative devices that have been shown to (i) improve chronic stroke patients' hip hiking in the swing phase and (ii) prolong the stance time on the paretic side and the step length on the non-paretic side [4]. There have been few such studies of patients who have suffered from an acute or subacute stroke, and it is not clear whether or how a robot should be prescribed during a patient's recovery process after stroke onset. Since the recovery of neuromuscular control in the gait during the acute and subacute stages influences the physical disability prognosis [5], it is very important to design interventions designed to improve patients' gait patterns from an early onset time point.

In the gait rehabilitation of stroke patients, an ankle-foot orthosis (AFO) and a knee-ankle-foot orthosis (KAFO) are commonly used for gait reconstruction [6]. An AFO is used to fix the ankle joint, and a KAFO is used to fix the knee and ankle joints. A KAFO is prescribed for patients who have experienced a severe stroke and cannot control knee instability during their standing or gait with an AFO [6]. When a patient's knee stability improves, it is common to transition from KAFO to AFO [6]. However, when a transition from a KAFO to an AFO is performed, it is extremely difficult to adjust the difficulty level for knee-joint movement, and an inappropriate adjustment (i.e., if knee instability remains) leads to the patient's mislearning of abnormal gait pattern(s) and ultimately to an asymmetrical gait. We hypothesized that the introduction of a robot with precise knee-joint movement adjustment during the transition from a KAFO to an AFO could optimize the degree of difficulty in the movement and prevent the mislearning of abnormal gait patterns, thus benefiting the acquisition of a symmetrical gait pattern.

Based on the above hypothesis, we introduced a gait rehabilitation program combining gait training with orthoses plus robot-assisted gait training for a patient who had suffered a subacute stroke and was unable to walk. We report that the patient made good progress and acquired a symmetrical gait pattern at the time of discharge.

## Case presentation

Case

**Figure 1:**
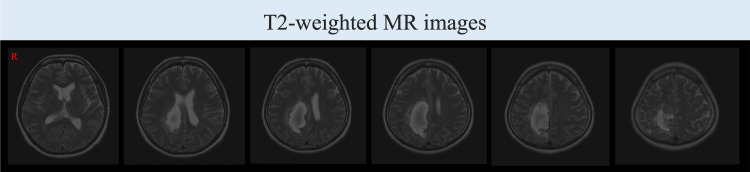
T2-weighted brain MR showed a high-signal response in a wide area centered on the vertex

A right-handed Korean woman in her 50s was taken to a hospital in an ambulance after experiencing a headache and difficulty in body movement. She was diagnosed with a right frontal subcortical hemorrhage (Figure 1) and had severe left upper-extremity and left-lower-extremity motor paralysis. The patient was transferred to our hospital for intensive rehabilitation 22 days after experiencing a stroke, following treatment at an acute care facility.

Written informed consent was obtained from the patient for publication of this case report and any accompanying images. A copy of the written consent is available for review by the editor-in-chief of this journal.

Course

On the day after the patient's admission to our hospital (23 days after the stroke onset), her Fugl-Meyer Assessment (FMA) score for the lower extremities (FMA-LE) [7] was 17/34 points, the FMA sensory score was 4/12, and sensorimotor deficits remained. The patient's Trunk Impairment Scale (TIS) score [8] was 10 points, and she scored 1 point on the Mini-Balance Evaluation Systems Test (Mini-BESTest) [9]. The Functional Ambulation Category (FAC) [10] was 0, and in the hospital, the patient always used a wheelchair (Table 1). The patient was the owner of a company and needed to return to work after discharge; she was thus strongly motivated to acquire an independent and symmetrical gait.

**Table 1:** The characteristics of the patient FAC: Functional Ambulation Category; FMA sensory score: sensory score of the Fugl-Meyer assessment; FMA-LE: lower-extremity motor subscale of the Fugl-Meyer assessment; MAS: modified Ashworth Scale; Mini-BESTest: Mini-Balance Evaluation Systems Test; TIS: Trunk Impairment Scale Reference: [7-11]

Factors	At admission (23 days from onset)	At 10 days (33 days from onset)	At discharge (69 days from onset)
FMA-LE, 0-34 points	17	26	28
FMA sensory score, 0-12 points	4	6	6
MAS, 0-5 points	2	2	1
Knee extensor strength, affected side, N	89.2	283.6	306.9
Knee extensor strength, unaffected side, N	226.5	325.5	360.8
TIS, 0-23 points	10	20	22
Mini-BESTest, 0-28 points	1	14	23
FAC, category 0-5	0	3	5
Maximum gait speed, m/s	−	0.83	1.25

Intervention

**Figure 2:**
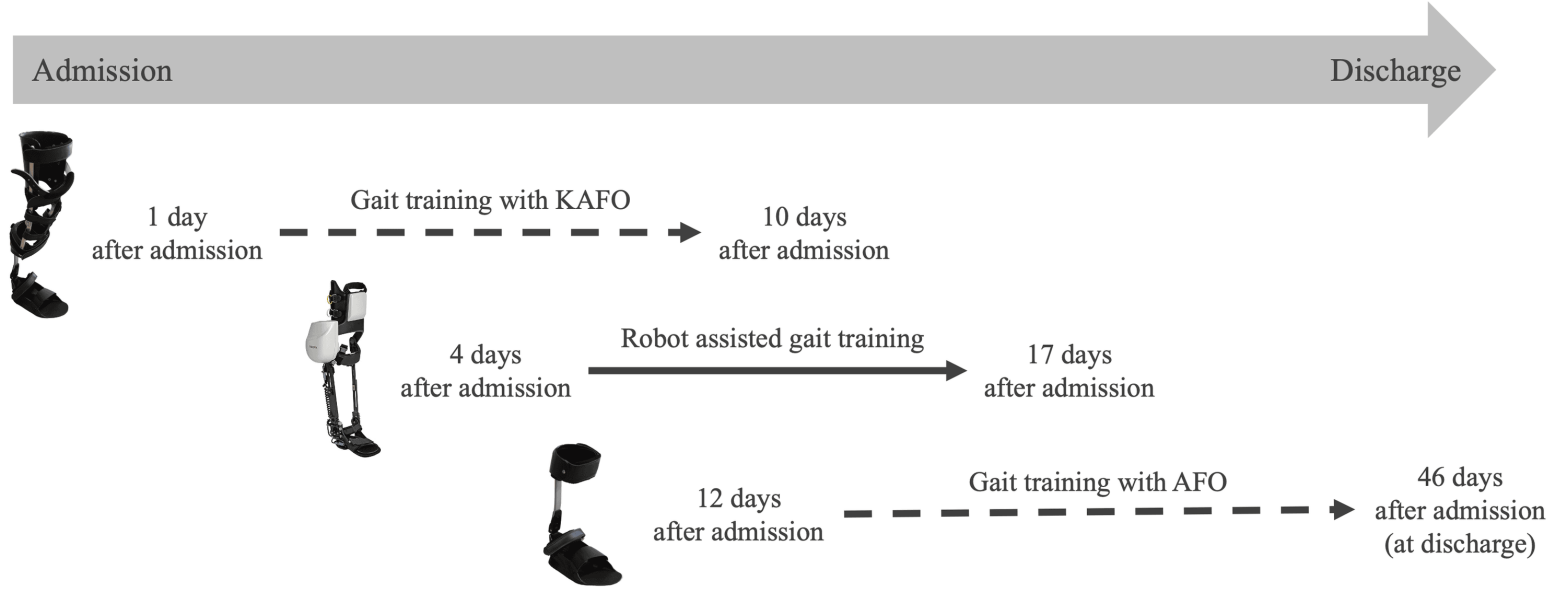
The stroke timeline and intervention procedure for the patient in this study AFO: ankle-foot orthosis; KAFO: knee-ankle-foot orthosis

Physical therapy was started the day after the patient's admission to our hospital. The gait training consisted of both gait training with orthoses and robot-assisted gait training, as depicted in Figure 2. A visual gait analysis confirmed that the patient had a gait abnormality due to a knee extension thrust pattern during the loading response phase and could barely walk with an AFO. Gait training with a KAFO was reported to promote the recovery of gait function in patients with knee instability [12], and we, therefore, provided gait training with a KAFO (RAPS, Tomei Brace, Aichi, Japan) for the patient in the early intervention phase (1-10 days after admission). For the intervention, the patient walked with the KAFO attached to her paralyzed left lower limb, and a physical therapist provided physical support for the patient's lower-limb movements in the stance and swing phase. As the patient's performance improved, the physical therapist gradually decreased the physical support.

As part of gait training with orthoses in general, when patients have regained stable control of the knee joint and are able to reproduce their KAFO gait with an AFO, a transition to an AFO (i.e., a cut-down) can be considered [12]. However, because of the greater mechanical difficulty involved in an AFO compared to a KAFO, mechanical differences affect the mislearning of knee movements during a gait, and in our clinical experience, a minor knee extension thrust pattern eventually remains. In the present patient, a visual gait analysis revealed the knee extension thrust pattern and its associated asymmetric gait pattern during her gait with an AFO (three days post-admission). Based on this observation, we considered it necessary to carefully adjust the task difficulty for the patient's knee joint in the cut-down. Robot-assisted gait training was therefore initiated in the middle intervention phase (4-17 days post-admission) as a preliminary step before transitioning to an AFO.

**Figure 3:**
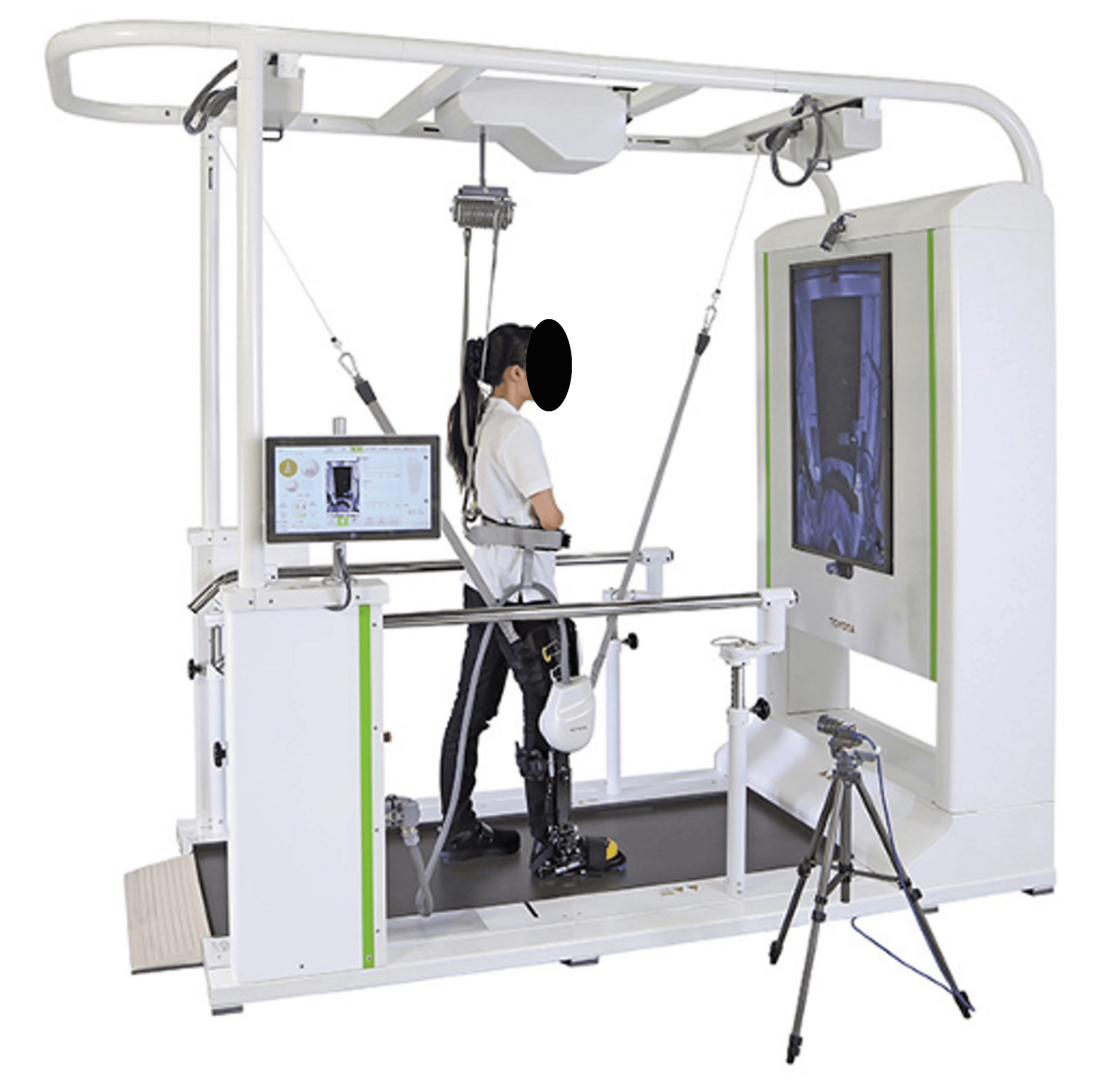
The gait exercise-assist robot Welwalk WW-1000 Image Credits: The figure is published with consent from Toyota Motor Corporation. Reference: [14]

The patient's intervention included the use of a walking-assist robot, the Welwalk WW-1000 (Welwalk, Toyota, Japan), which was used for the patient's intervention [13,14]. As shown in Figure 3, the Welwalk is a walking-assist robot comprising a KAFO robot, a low-floor treadmill, a safety suspension harness, a robot relief system, a front-facing monitor for the patient, and a control panel for the therapist. In the intervention, the patient practiced walking on the treadmill while wearing the knee-ankle-foot robotic device on her paralyzed lower limb. The Welwalk is characterized by its high feedback (visual feedback via the front monitor and auditory feedback via sound) and precise adjustability (e.g., lower-limb assist during the swing and stance phase), which enables gait training based on the motor learning theory. The Welwalk KAFO robot has precise adjustability, and it appropriately assists in the knee joint's flexion during the swing phase and the knee joint's extension during the stance phase. For the KAFO robot's assistance, the amount of knee-extension assistance was adjusted to the minimum level that prevents knee bending during the stance phase, and the amount of knee-flexion assistance was adjusted to the minimum level that allows the user to swing her leg. During the present patient's intervention, coaching by a physical therapist and visual and auditory feedback were used to adjust the gait pattern to avoid the appearance of compensatory movements.

A final intervention phase (12-46 days after admission) using gait training with an AFO (RAPS, Tomei Brace, Japan) was performed. During this phase, the patient was able to walk independently without the assistance of a physical therapist, and only a slight knee extension thrust pattern was observed during her gait. Gait training with the AFO was thus conducted under the supervision of a physical therapist while the patient also used performance-based gait aids (e.g., side cane, quad cane, and T-cane). The patient was eventually able to perform outdoor walking and stair climbing without the AFO and gait aids.

All gait training was administered in 40-minute sessions that consisted of approximately 30 minutes of gait training and short breaks as needed. The patient was also offered 2-3 hours/day of a general rehabilitation program (e.g., range of motion exercise, muscle-strengthening exercise, training for activities of daily living (ADLs)) in addition to the gait training.

Measurements

The patient's clinical evaluations were performed at the time of her admission to our hospital, 10 days after admission, and at discharge. The patient was evaluated using the FMA to measure the severity of sensory disturbance (FMA-sensory score) and lower-extremity motor paralysis (FMA-LE) [7]. Muscle spasticity in the ankle plantar flexor was assessed with the modified Ashworth Scale (MAS) [11], and the knee extension strength was measured as an indicator of lower limb muscle strength [15]. Functional performance was evaluated using the TIS [8], the Mini-BESTest [9], and the FAC [10]. Additionally, her maximum gait speed was determined by timing as she traversed a 10-meter walkway using a stopwatch [16].

A three-dimensional (3D) gait analysis was performed for 10 days post-admission and at the time of discharge with the use of a 3D motion capture system (Kinema Tracer, Kissei Comtec, Japan) to evaluate the pattern of knee movement and spatiotemporal gait asymmetry. The details of all measurement protocols are as follows. First, reflective markers were attached to the patient's body at 12 locations (the acromion, iliac crest, hip joint, lateral femoral epicondyle, lateral malleolus, and fifth metatarsal head on both sides). The measurement task was the gait on a treadmill, with one handrail grasped. In the analysis of knee-joint movement, we identified movement patterns corresponding to the normal knee pattern, buckling-knee pattern, extension thrust pattern, and stiff-knee pattern, based on the report by De Quervain et al. [17]. For the analysis of gait asymmetry, we utilized the values of the swing times and the step lengths derived from the 3D gait analysis. The asymmetry magnitudes for swing time and step length were calculated as the ratio of the left and right values, with the larger value serving as the numerator regardless of the affected side [3]. A ratio of 1.0 represents complete symmetry. As the cut-off values of each variable are reported, the swing time (temporal) symmetry ratio is 1.06, and the step length (spatial) symmetry ratio is 1.08 [3].

Results

Following the interventions for our patient, some extent of recovery was observed regarding the weakness of her left lower extremity (from the FMA-LE score of 17 to 28), her trunk function (from the TIS score of 10 to 22), and her balance performance (from the Mini-BESTest score of 1 to 23 points) at discharge (Table 1). As a result, the patient was able to walk independently, and her FAC reached 5 points (Table 1). Her gait speed also improved to 1.25 m/s.

**Figure 4:**
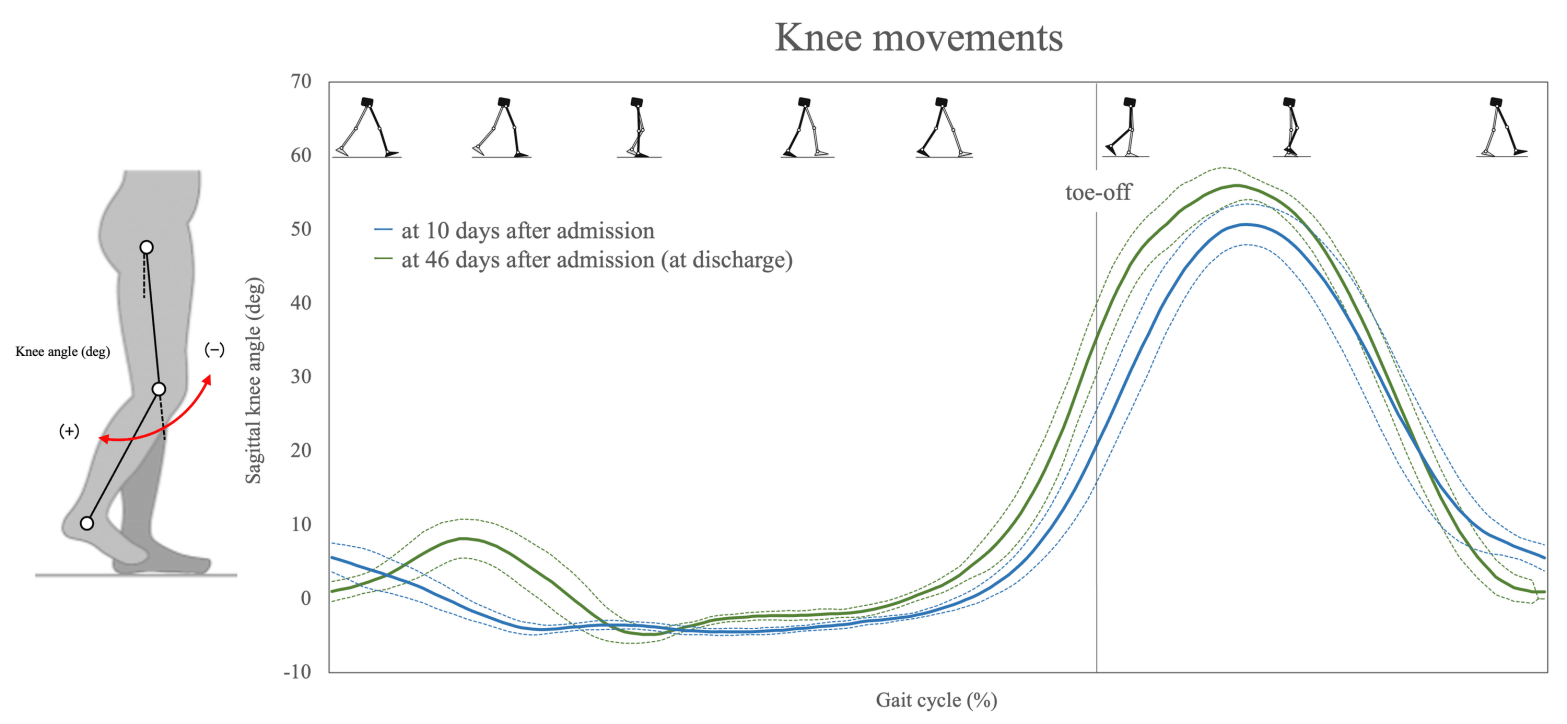
Changes in the patient's knee joint movement during gait Solid line: the mean value of recorded data; dashed lines: the standard deviation of the recorded data.

**Figure 5:**
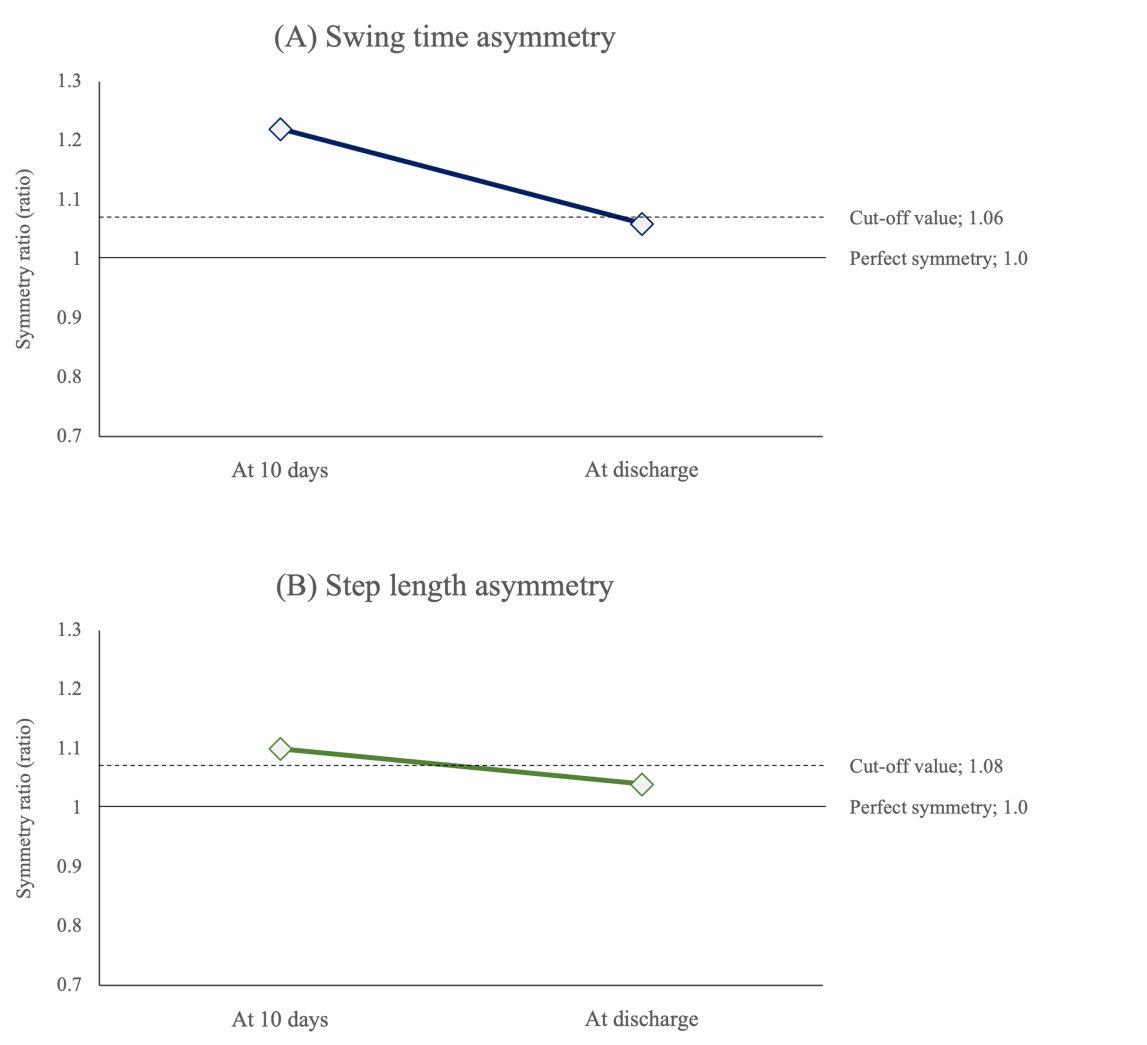
Changes in the patient's swing time (A) and step length asymmetries (B). Each variable improved to symmetry at the patient's discharge

The patterns of the patient's knee movement showed the extension thrust pattern 10 days after admission but improved to the normal knee pattern at discharge with the appearance of knee flexion in the loading response (Figure 4). Regarding the patient's gait pattern, the temporal symmetry ratio changed from 1.22 to 1.06, and the spatial symmetry ratio changed from 1.1 to 1.04, leading to a symmetrical gait (Figure 5).

## Discussion

We conducted combined gait training with orthoses and robot-assisted gait training for a post-stroke patient from the early intervention phase. At the time of her discharge, the patient had acquired a symmetrical gait, indicating that this combined intervention protocol could be effective as gait training for abnormal gait patterns in subacute stroke patients. Gait asymmetry is a frequently observed gait disorder in post-stroke patients. The cut-off values for temporal asymmetry and spatial asymmetry in an earlier study were 1.06 and 1.08, respectively. Our patient was observed to have gait asymmetry with a temporal symmetry ratio of 1.23 and a spatial symmetry ratio of 1.1 at 10 days after admission. In the present patient, we speculate that the extension thrust pattern during the loading response phase was the cause of the asymmetry pattern.

The extension thrust pattern is a compensatory movement to passively stabilize the knee by hyperextension of the knee joint in an individual with knee-control dysfunction, based on decreased muscle activity of the knee extensors and ankle plantar flexors [18]. This stance-phase problem also affects the swing phase, and an effortful limb swing to compensate for forward propulsion modulates the swing time and the step length [18]. In fact, such an abnormal gait pattern is associated with a decrease in gait speed and causes gait asymmetry [2,3]. Based on the above, we considered that gait training for instability of the knee joint by extension knee thrust was necessary to improve our patient's gait performance.

Gait training with a KAFO is a useful method for improving an individual's gait performance and ability to engage in ADLs [12]. Maeshima et al. reported that intensive standing and gait training using a KAFO for patients with severe hemiplegia resulted in improved ability to perform ADLs and shorter hospitalization stays [19]. Abe et al. also reported that gait training with a KAFO for patients with severe hemiplegia and knee instability facilitated improvement in gait ability [12]. A transition from a KAFO to an AFO is frequently conducted as a patient's gait performance improves, but there are no clear criteria for this transition. One problem with the transition period is that structural differences between KAFOs and AFOs cause difficulty in fitting the task difficulty of the knee-joint movement, and we have observed the appearance of abnormal gait patterns during this period.

The Welwalk robot that we introduced in the present patient's transition period has precise adjustability of knee-joint movement based on the motor learning theory [13]. Robotic assistance of lower-limb movements causes a modulation of the muscle activity of the lower limb during the gait cycle, with a certain learning effect [20]. Our patient had spatiotemporal gait asymmetry 10 days after her admission, but at discharge, she had improved to a symmetrical gait. The majority of stroke patients have residual gait asymmetry even after discharge from a hospital, due to a background of insufficient rehabilitation for gait asymmetry [2]. The change in our patient's gait pattern case could be considered a clinically meaningful improvement, and the results of other studies support this [4,13]. Our patient's course suggests that the combination of robot-assisted gait training with gait training with orthoses for subacute stroke patients may prevent the mislearning of gait movements and contribute to the acquisition of a symmetrical gait pattern.

This study has some limitations. It is a retrospective case report, and thus the evidence could be insufficient. Further single-case studies should be conducted and eventually validated in a population with a prospective study design. In addition, our patient's knee instability during gait at the early intervention phase and during the transition from a KAFO to an AFO was assessed based on a visual gait analysis, because a 3D gait analysis required the patient to walk independently on the treadmill. Since bias exists in visual gait analysis, it is necessary to devise a method for a quantitative evaluation (e.g., a 3D gait analysis of gaits on level ground). Finally, we suspected that the cause of our patient's gait asymmetry was instability of the knee, but other factors might have been involved. Nevertheless, the improvement in the patient's abnormal knee joint movement and gait asymmetry over time may support our initial hypothesis. Despite the limitations described above, this case report provides useful information for decision-making in gait rehabilitation, as it describes the clinical application of a rehabilitation robot in a patient's recovery process after a stroke.

## Conclusions

In conclusion, our patient's case suggests that combined robot-assisted gait training and gait training with orthoses may prevent subacute stroke patients' mislearning of gait movements and contribute to the acquisition of a symmetrical gait pattern.
